# Multi-walled carbon nanotubes promote the accumulation, distribution, and assimilation of ^15^N-KNO_3_ in *Malus hupehensis* by entering the roots

**DOI:** 10.3389/fpls.2023.1131978

**Published:** 2023-03-09

**Authors:** Junyuan Shi, Mi Xun, Jianfei Song, Jiaqi Li, Weiwei Zhang, Hongqiang Yang

**Affiliations:** College of Horticulture Science and Engineering, Shandong Agricultural University, Tai’an, Shandong, China

**Keywords:** multi-walled carbon nanotubes, nitrogen metabolism, nitrate transporter; ^15^N, *Malus*, agricultural nanotechnology

## Abstract

**Introduction:**

Multi-walled nanotubes (MWCNTs) consist of multiple rolled layers of graphene. Nitrogen plays an important role in apple growth. The effect of MWCNTs on nitrogen utilization in apple needs to be further investigated.

**Methods:**

In this study, the woody plant *Malus hupehensis* seedlings were used as plant materials, the distribution of MWCNTs in the roots was observed, and the effects of MWCNTs on the accumulation, distribution, and assimilation of nitrate by the seedlings were explored.

**Results:**

The results showed that MWCNTs could penetrate the roots of *Malus hupehensis* seedlings, and the 50, 100, and 200 µg·mL^-1^ MWCNTs significantly promoted the root growth of seedlings, increased root number, root activity, fresh weight, and nitrate content of seedlings, and also increased nitrate reductase activity, free amino acid, and soluble protein content of roots and leaves. ^15^N tracer experiments indicated that MWCNTs decreased the distribution ratio of ^15^N-KNO_3_ in *Malus hupehensis* roots but increased its distribution ratio in stems and leaves. MWCNTs improved the utilization ratio of ^15^N-KNO_3_ in *Malus hupehensis* seedlings, with the values being increased by 16.19%, 53.04%, and 86.44% following the 50, 100, and 200 µg·mL^-1^ MWCNTs, respectively. The RT-qPCR analysis showed that MWCNTs significantly affected the expression of genes (*MhNRTs*) related to nitrate uptake and transport in roots and leaves, and *MhNRT1.4*, *MhNRT1.7*, *MhNRT1.8*, *MhNRT2.1*, *MhNRT2.5*, and *MhNRT2.7* were notably up-regulated in response to 200 µg·mL^-1^ MWCNTs. Raman analysis and transmission electron microscopy images indicated that MWCNTs could enter the root tissue of *Malus hupehensis* and were distributed between the cell wall and cytoplasmic membrane. Pearson correlation analysis showed that root tip number, root fractal dimension, and root activity were the main factors affecting root uptake and assimilation of nitrate.

**Conclusions:**

These findings suggest that MWCNTs promoted root growth by entering the root, stimulated the expression of *MhNRTs*, and increased NR activity, thereby enhancing the uptake, distribution, and assimilation of nitrate by root, and ultimately improved the utilization of ^15^N-KNO_3_ by *Malus hupehensis* seedlings.

## 1 Introduction

Nanotechnology is becoming one of the most pioneering and promising technology for transforming conventional food and agriculture industries with the research and application of nanomaterials (Ashraf et al., 2021). Nitrogen is an essential nutrient for plant growth and crop productivity, the effect of nanomaterials on plant nitrogen uptake and utilization is an important basis for the rational application of nanomaterials in agricultural production.

Carbon nanotubes (CNTs) are one of the most produced nanomaterials worldwide, including single-walled carbon nanotubes (SWCNTs) and multi-walled carbon nanotubes (MWCNTs) (Zhai et al., 2015). CNTs are widely used not only in wastewater treatment, biomedicine, and environmental protection, but also have important prospects in agriculture, because of their unique features, such as strong adsorption capacity, large specific surface area, excellent photocatalysis, and infrared absorption (Ali et al., 2021). It has been reported that MWCNTs can increase the calcium and iron content in maize seedlings (Tiwari et al., 2013), promote nodulation and nitrogen fixation in the legume of Lotus japonicus (Yuan et al., 2017), improve the growth and yield of wheat and rice (Joshi et al., 2018; Joshi et al., 2020), and also promote maize seedlings growth by coordinating carbon and nitrogen metabolism (Hu et al., 2021), etc. However, these studies are mainly limited to the response of some herbaceous crops’ physiological and agronomic traits to MWCNTs.

Nitrogen is a macroelement necessary for plant growth, ${\text{NO}}_{3}^{-}$ and ${\text{NH}}_{4}^{+}$ are the two most common sources of nitrogen that can be absorbed and used by plants. Apple (*Malus domestica* Borkh.) is an important woody cash crop that prefers to utilize ${\text{NO}}_{3}^{-}$ (Li et al., 2013). After the external nitrate enters the root through nitrate transporters (*NRTs*), it can be left in roots or transported to stems and leaves, and then assimilated into amino acids and proteins through the actions of a series of enzymes (Zhao et al., 2018). Nitrate reductase (NR) is a rate-limiting enzyme that regulates nitrogen metabolism and assimilation, and MWCNTs can regulate maize carbon and nitrogen metabolism and promote growth by regulating enzymes related to carbon and nitrogen metabolism including NR (Erdal, 2019; Hu et al., 2021).

*Malus hupehensis* Rehd. is an apple rootstock, the apple branch-derived biochar powder with a particle size of less than 1 mm can promote root growth, improve root activity and NR activity, promote the nitrate uptake and utilization of seedlings, and significantly increase leaf photosynthetic rate and biomass after it was applied to the seedlings root zone (Yan et al., 2014; Cao et al., 2019; Shi et al., 2022). MWCNTs are exogenous carbon with a smaller particle size than biochar powder, which can penetrate the seed coat of corn, barley, and soybean, enhance the expression of aquaporin genes, and then promote the water absorption and germination of seeds (Lahiani et al., 2013); MWCNTs can also enter the broccoli roots, change the permeability of the cytoplasmic membrane, enhance the transduction performance of aquaporins, and improve the water uptake capacity of the roots, thereby alleviating salt stress (Martinez-Ballesta et al., 2016). The uptake of mineral nutrients by roots is accompanied by water; therefore, MWCNTs may also affect the uptake of mineral nutrients by roots in this way.

Based on the above, the woody plant *Malus hupehensis* seedlings were used as the test material in this study. We explored the changes in microstructural (transmission electron microscopy observation and Raman spectroscopy analysis), morphological (root architecture), physiological (^15^N isotope analysis, nitrate accumulation, enzymes activity, assimilation products), and molecular (transcript levels of representative genes related to nitrate uptake and transport). The goals of this study were to determine (1) if MWCNTs promote plants growth, (2) if MWCNTs promote the accumulation, utilization, and assimilation of nitrate in *Malus hupehensis*, (3) if MWCNTs enter the plant root and transit to the aboveground. This study provides new insights into the interaction of MWCNTs with plant nutrient uptake and lays the foundation for the development of precision agriculture.

## 2 Materials and methods

### 2.1 Characterization of MWCNTs

MWCNTs were purchased from Chengdu Organic Chemistry Co. Ltd., Chinese Academy of Sciences (Chengdu, China), basic properties are listed in the **Supplementary Table 1**. Transmission electron microscopy (TEM) (JEOL-1400Plus, Japan) and Roman spectra (Horiba Scientific LabRAM HR Evolution, France) were used to characterize the morphology and structure of MWCNTs (**Supplementary Figure 1**).

### 2.2 Plant cultivation and treatments

Experiments were conducted at the National Research Center of Apple Engineering and Technology, Shandong Agriculture University, Tai’an (36°10′N, 117°07′E), China, from October 2020 to May 2021. The full *Malus hupehensis* seeds were soaked in water for 24 h before being mixed with thoroughly washed fine sand (1:1, v/v) at 4°C stratifications for 40–60 days. The germinated seeds were cultivated in plastic pots filled with soil that was mixed with turf, perlite, and vermiculite (3:1:1, v/v) in the greenhouse until they produced two leaves. Then, the consistent seedlings were transferred to black glass culture flasks and were secured with perforated aluminum foil, 2 seedlings per bottle. When the seedlings were cultured with Hoagland nutrient solution (5 mM KNO_3_, 1 mM CaCl_2_, 1 mM KH_2_PO_4_, 1 mM MgSO_4_, 0.1 mM FeSO_4_·7H_2_O, 0.1 mM Na_2_-EDTA·2H_2_O, 50 µM MnSO_4_·H_2_O, 50 µM H_3_BO_3_, 0.05 µM CuSO_4_·5H_2_O, 0.5 µM Na_2_MoO_4_·2H_2_O, 15 µM ZnSO_4_·7H_2_O, 2.5 µM KI, 0.05 µM CoCl·6H_2_O) (Liu et al., 2021) to 5–6 leaves, the nutrient solution was replaced with K^15^NO_3_ nutrient solution and MWCNTs treatment was performed.

K^15^NO_3_ nutrient solution was to replace 5 mM KNO_3_ in the above nutrient solution with “4 mM KNO_3_ + 1 mM K^15^NO_3_”, K^15^NO_3_ at abundances of 10.15% was purchased from Shanghai Research Institute of Chemical Industry (Shanghai, China). MWCNTs were added to the K^15^NO_3_ nutrient solution at different concentrations to make suspensions: 0 μg·mL^-1^ (Control), 50 μg·mL^-1^, 100 μg·mL^-1^, and 200 µg·mL^-1^ (Khodakovskaya et al., 2011), and then the suspensions were sonicated (100 W, 40 Hz) for 30 minutes before seedlings exposure. Twelve replicates were established for each treatment, three of which were used without isotopic labeling for natural abundance determination. The MWCNTs suspension was stirred every 12 h by a glass rod and replaced with freshly prepared MWCNTs suspension every 3 days. The cultivated seedlings were conducted in a greenhouse with natural sunlight, average day/night temperatures of 24/17°C, and relative humidity of 50–60%. Seedlings were destructively sampled after 30 days, some samples were used for the determination of fresh weight, root architecture, root activity, nitrate content, enzyme activities, free amino acids, and soluble protein content, and some samples were dried to a constant weight at 80°C for ^15^N abundance and total nitrogen content determination, and the rest were stored at –80°C for total RNA extraction.

### 2.3 Root architecture parameters and root activity

Roots of seedlings were placed in a tray with water to obtain the relevant images using a scanner (ScanMaker i800 plus, China). The total root length, root surface area, root tip number, root fractal dimension, and other root architecture parameters were determined using a Wanshen LA-S series plant image analyzer (China). The reduction of triphenyl tetrazolium chloride (TTC) was used to determine the root activity of the seedlings (Zhao et al., 2002).

### 2.4 Nitrate and total nitrogen content and stable isotope analysis

The nitrate content was determined using salicylic acid colorimetry. Weigh fresh samples were homogenized in deionized water, then the mixture was incubated at 45°C for 1 h and centrifuged, and the supernatant was mixed with the salicylic acid-concentrated sulfuric acid mixture and reacted for 20 min at room temperature, the pH of the mixture was adjusted to 12 and the absorbance was measured at 410 nm (Patterson et al., 2010).

The total N content of the seedlings was determined using a kjeldahl apparatus (K9860, China), and the abundance of ^15^N was determined using a mass spectrometer (MAT-251, Germany). The calculations of the ^15^N isotope referred to Shi et al. (2022) as follows:


$$\text{Ndff }(\text{nitrogen derived from fertilizer})\;(\%)\;=\frac{\text{abundance of }{\;}^{15}\text{N in plant }-\text{natural abundance of }{\;}^{15}\text{N}}{\text{abundance of }{\;}^{15}\text{N in fertilizer }-\text{natural abundance of }{\;}^{15}\text{N}}\times 100\%$$

$${\;}^{15}\text{N absorbed by each organ from fertilizer }(\text{mg})\;=\text{Ndff}\;(\%)\;\times \;\text{organ total nitrogen}(\text{mg})$$

$${\;}^{15}\text{N distribution ratio}(\%)\;=\frac{{\;}^{15}\text{N absorbed by each organ from fertilizer }(\text{mg})}{\text{total }{\;}^{15}\text{N absorbed by plant from fertilizer }(\text{mg})}\times 100\%$$

$${\;}^{15}\text{N utilization ratio}(\%)\;=\frac{\text{Ndff }\times \text{total }{\;}^{15}\text{N of organs }(\text{mg})}{{\;}^{15}\text{N fertilization }(\text{mg})}\times 100\%$$

### 2.5 NR activity, free amino acids, and soluble protein content

NR (EC1.6.6.1) activity was determined referred to the method of Hu et al. (2016). The free amino acid and soluble protein contents were determined using the ninhydrin method and the Bradford method, respectively (Hu et al., 2021).

### 2.6 Transmission electron microscopy observation and Raman scattering spectroscopy analysis

TEM observation of MWCNTs: MWCNTs (0.01 g) was stirred into 50 mL of anhydrous ethanol, and the mixture was sonicated (100 W, 40 Hz) for 30 minutes to form the MWCNTs dispersion, then, a small amount of MWCNTs dispersed droplets were added on the copper mesh and observed under a TEM.

Seedling samples preparation and TEM observation referring to the method of Du et al. (2016): Seedlings samples were thoroughly washed using deionized water, prefixed in 2.5% (v/v) glutaraldehyde for 24 h, and then soaked in PBS buffer for 2 h, prefixed in 1% osmium tetroxide for 4.5 h and soaked in PBS buffer for 2 h, dehydrated in a graded series of ethanol (45%, 55%, 75%, 85%, 95%, and 100%) and then embedded in epoxy resin (DMP-30). Finally, the samples were dried and sliced on the machine for observation.

Raman spectroscopy: The dried MWCNTs or plant powder were detected at an excitation wavelength of 633 nm (Khodakovskaya et al., 2013).

### 2.7 Total RNA extraction and RT-qPCR analysis

Total RNA was extracted from *Malus hupehensis* using RNAprep Pure Plant Kit (Tiangen Biotech, China). Then, 1 µg of the total RNA was used to synthesize the cDNA using the HiScript^®^ III RT SuperMix for qPCR (+ gDNA wiper) (Vazyme, China).

RT-qPCR was performed in a LightCycler^®^ 96 (Roche) using the Taq Pro Universal SYBR qPCR Master Mix (Vazyme, China) under the following conditions: 45 cycles of 95°C for 5 s and 60°C for 30 s. All primers used for RT-qPCR are listed in **Supplementary Table 2**. Three biological and technical replications were assayed and the data were calculated using the 2^-ΔΔCt^ method.

### 2.8 Statistical analysis

Statistical analyses were performed after all data were checked for normality (Shapiro-Wilk test) and homogeneity of variance (Levene’s test). Data were analyzed using one-way factorial analysis of variance (ANOVA), and significant differences among means between the treatments were compared using Duncan’s multiple range test at *P*< 0.05 in SPSS 23.0 (IBM Corporation, USA). Microsoft Excel 2016 (Microsoft Corporation, USA) and Origin 2021 (Originlab Corporation, Northampton, MA, USA) were used for chart drawing.

## 3 Results

### 3.1 MWCNTs increased nitrate content and biomass in *Malus hupehensis* seedlings

**Figure 1:**
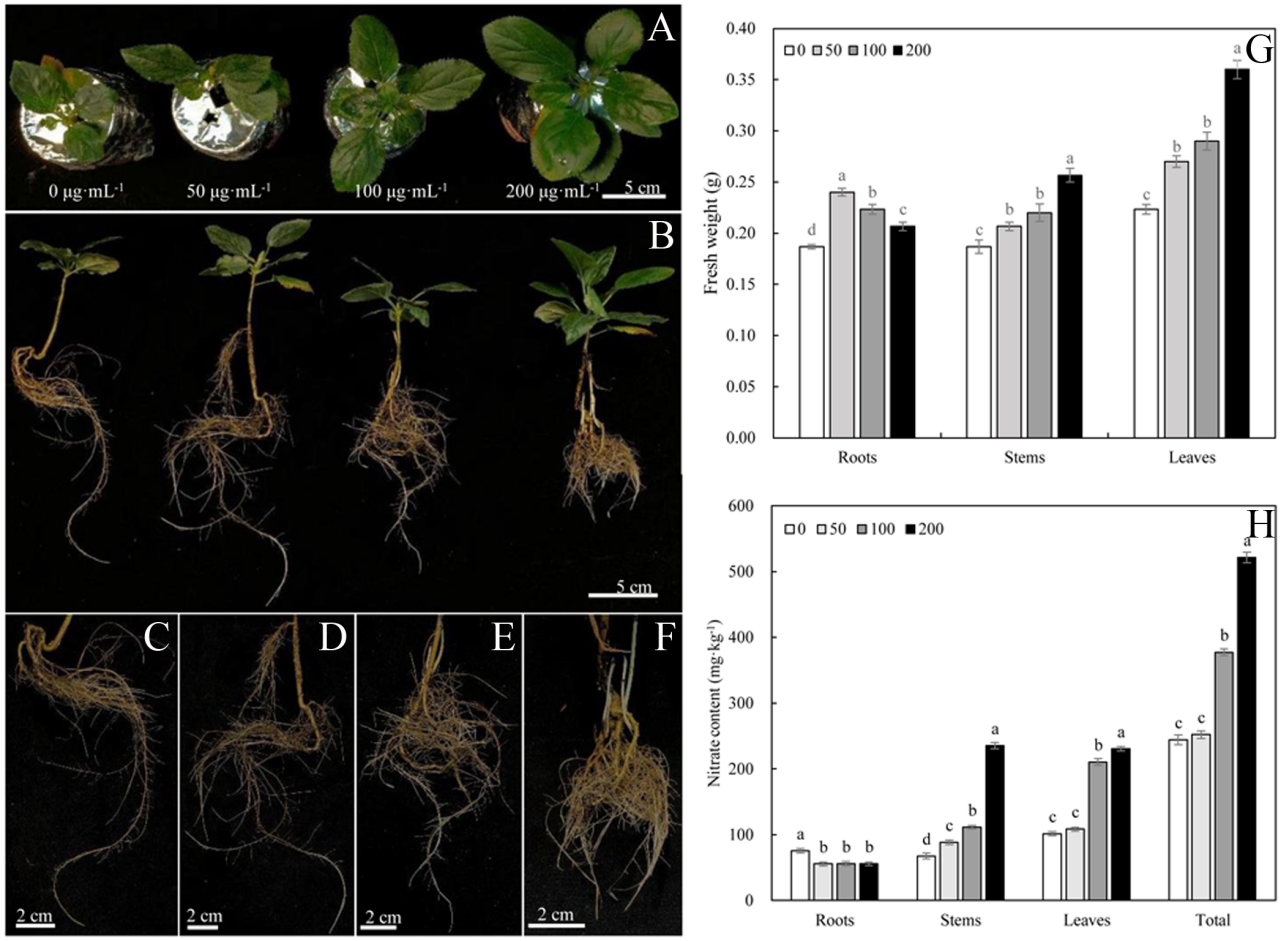
Phenotypes of leaves **(A)**, roots **(C–F)**, and whole plant **(B)**, fresh weights of roots, stems, and leaves **(G)** and nitrate content **(H)** of *Malus hupehensis* seedlings under the 0, 50, 100, and 200 µg·mL^-1^ MWCNTs treatments for 30 days. Different lowercase letters indicate significant difference at *P*< 0.05, and error bars represent the standard deviation of the mean (One-way ANOVA, n=6).

The phenotype of Malus hupehensis responded strongly to MWCNTs (**Figures 1A–F**). MWCNTs at 50, 100, and 200 µg·mL^-1^ significantly increased the fresh weight of roots, stems, and leaves of seedlings (*P*< 0.05), however, the increase of root fresh weight gradually decreased with the increasing concentration of MWCNTs, while the increase of stem and leaf fresh weight gradually increased. 50 µg·mL^-1^ MWCNTs increased the root fresh weight by 26.32%, and 200 µg·mL^-1^ MWCNTs increased the stem and leaf fresh by 36.84% and 63.64%, respectively (**Figure 1G**).

The root nitrate content of *Malus hupehensis* seedlings was significantly decreased with the application of MWCNTs (*P*< 0.05), while the stem, leaf, and whole plant of seedling nitrate content were increased. The whole plant seedling nitrate content increased with the increasing concentration of MWCNTs, with the values being increased by 3.27%, 54.56%, and 113.58% following 50, 100, and 200 µg·mL^-1^ MWCNTs treatments, respectively (**Figure 1H**).

### 3.2 Effect of MWCNTs on root architecture parameters and root activity of *Malus hupehensis* seedlings

**Figure 2:**
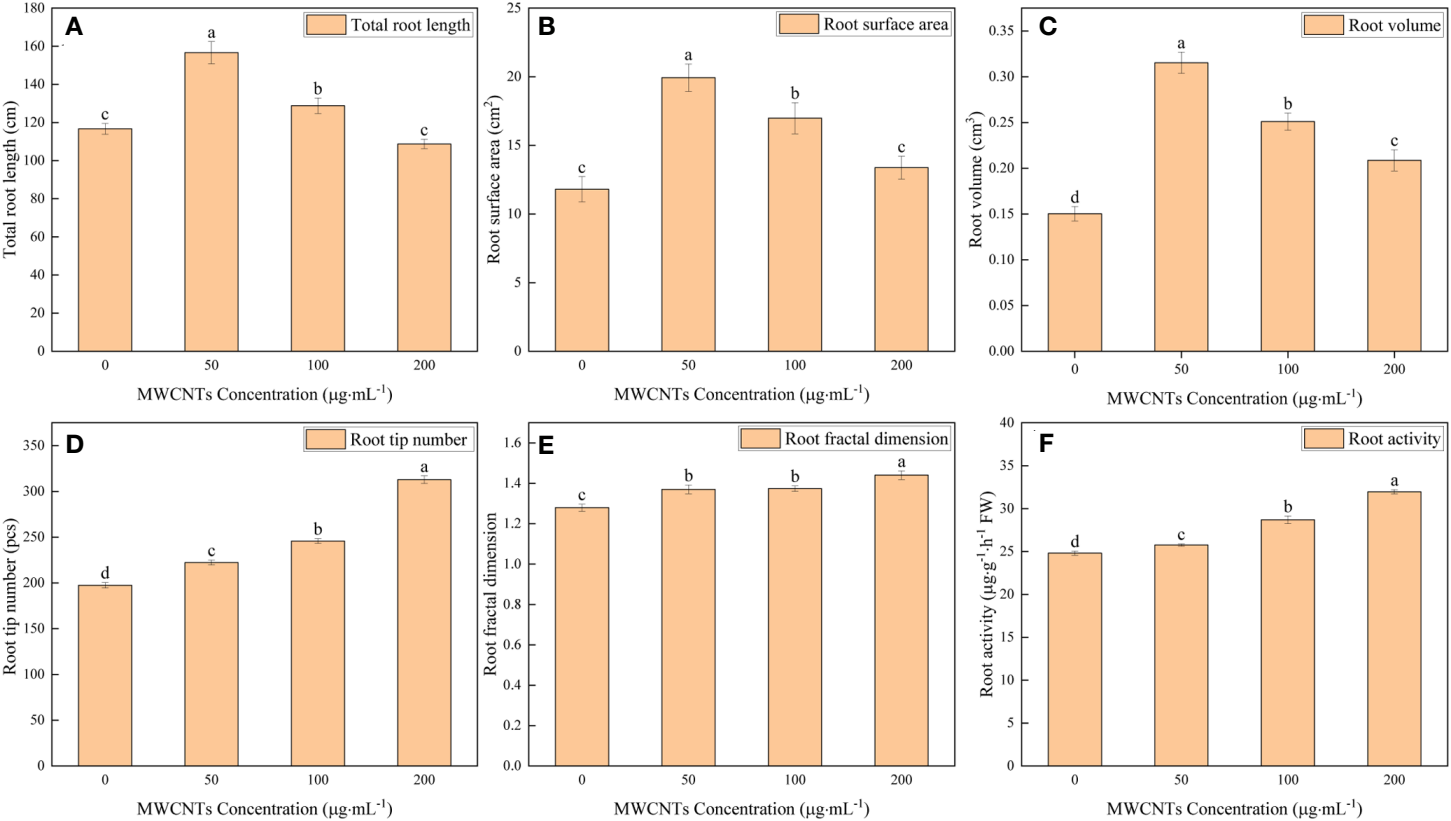
Total root length **(A)**, root surface area **(B)**, root volume **(C)**, root tip number **(D)**, root fractal dimension **(E)**, and root activity **(F)** of *Malus hupehensis* seedlings under the 0, 50, 100, and 200 µg·mL^-1^ MWCNTs treatments for 30 days. Different lowercase letters indicate significant difference at *P*< 0.05, and error bars represent the standard deviation of the mean (One-way ANOVA, n=6).

The 0, 50, 100, and 200 µg·mL^-1^ MWCNTs treatments affected the root morphology of *Malus hupehensis* seedlings (**Figures 1C–F**), and increased the total root length, root surface area, root volume, root tip number, and root fractal dimension (**Figures 2A–E**). Among them, the total root length, root surface area, and root volume increased the most under 50 µg·mL^-1^ MWCNTs treatment, with the values being increased by 34.24%, 68.76%, and 113.33%, respectively, while the increase was reduced following 100 and 200 µg·mL^-1^ MWCNTs treatments. The root tip number and root fractal dimension increased with the increasing concentration of MWCNTs, with the values being increased by 58.39% and 12.50% under 200 µg·mL^-1^ MWCNTs, respectively.

Moreover, MWCNTs also significantly improved the root activity of *Malus hupehensis* (*P*< 0.05) (**Figure 2F**), and the increases were 3.83%, 15.64%, and 28.82% following 50, 100, and 200 µg·mL^-1^ MWCNTs treatments, respectively.

### 3.3 Effects of MWCNTs on the accumulation, utilization, and distribution of ^15^N-KNO_3_ in *Malus hupehensis* seedlings

**Figure 3:**
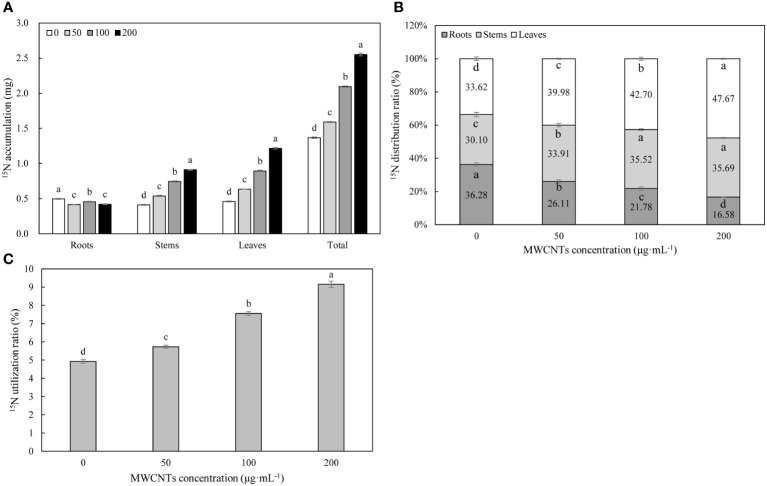
^15^N-KNO_3_ accumulation **(A)** and distribution ratio **(B)** in each organ, and ^15^N-KNO_3_ utilization ratio **(C)** in the whole plant of *Malus hupehensis* seedlings under the 0, 50, 100, and 200 µg·mL^-1^ MWCNTs treatments for 30 days. Different lowercase letters indicate significant difference at *P*< 0.05, and error bars represent the standard deviation of the mean (One-way ANOVA, n=6).

^15^N isotope labeling results revealed that MWCNTs significantly affected the accumulation, utilization, and distribution of ^15^N-KNO_3_ in different organs of *Malus hupehensis* (*P*< 0.05) (**Figure 3**). MWCNTs decreased the accumulation of ^15^N-KNO_3_ in roots, but significantly increased the accumulation of ^15^N-KNO_3_ in stems, leaves, and the whole plant (*P*< 0.05), 50, 100, and 200 µg·mL^-1^ MWCNTs treatments increased the accumulation of ^15^N-KNO_3_ in whole plant of seedlings by 16.06%, 53.28%, and 86.13%, respectively (**Figure 3A**).

MWCNTs significantly reduced the distribution ratios of ^15^N-KNO_3_ in the *Malus hupehensis* roots and increased its distribution ratios in the stems and leaves (*P*< 0.05). The distribution ratios of ^15^N-KNO_3_ in the roots were decreased by 28.03%, 39.97%, and 54.13% following 50, 100, and 200 µg·mL^-1^ MWCNTs treatments, respectively, while the values were increased by 12.66%, 18.01%, and 18.54% in stems, respectively, and increased by 18.92%, 27.01%, and 41.79% in leaves, respectively (**Figure 3B**).

MWCNTs also significantly increased the utilization ratio of ^15^N-KNO_3_ in *Malus hupehensis* seedlings (*P*< 0.05), and increased by 16.19%, 53.04%, and 86.44% following 50, 100, and 200 µg·mL^-1^ MWCNTs treatment, respectively (**Figure 3C**).

### 3.4 Effect of MWCNTs on NR activity, free amino acid, and soluble protein contents in *Malus hupehensis* seedlings

**Figure 4:**
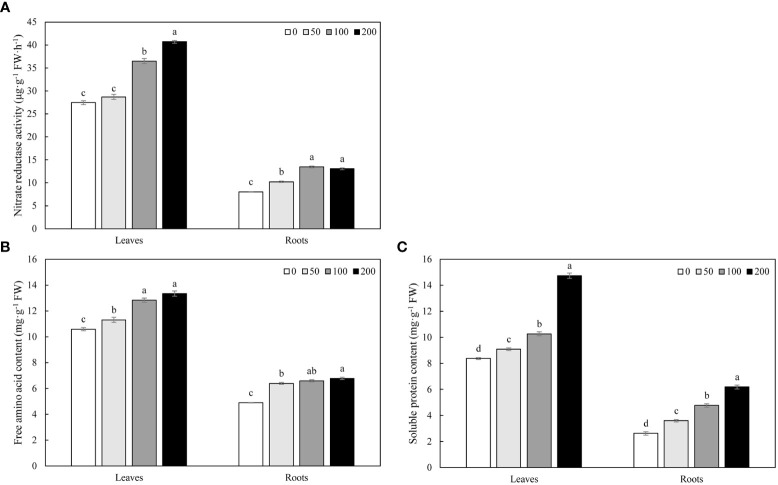
NR activity **(A)**, free amino acid **(B)**, and soluble protein content **(C)** in roots and leaves of *Malus hupehensis* seedlings under the 0, 50, 100, and 200 µg·mL^-1^ MWCNTs treatments for 30 days. Different lowercase letters indicate significant difference at *P*< 0.05, and error bars represent the standard deviation of the mean (One-way ANOVA, n=6).

MWCNTs increased the NR activity in roots and leaves of *Malus hupehensis* seedlings, 50, 100, and 200 µg·mL^-1^ MWCNTs significantly increased the NR activity in roots by 27.43%, 67.83%, and 62.72%, respectively (*P*< 0.05), while the increase of NR activity in leaves only reached a significant level under 100 and 200 µg·mL^-1^ MWCNTs treatments (*P*< 0.05) (**Figure 4A**).

MWCNTs significantly increased the contents of free amino acid and soluble protein in roots and leaves of *Malus hupehensis* seedlings with the increasing concentration of MWCNTs (*P*< 0.05). The contents of free amino acid in roots were increased by 30.67%, 34.56%, and 38.65%, respectively, and the values were increased by 6.80%, 21.15%, and 25.97% in leaves, respectively. In addition, MWCNTs also significantly increased the soluble protein contents in roots by 37.02%, 81.68%, and 136.26%, respectively (*P*< 0.05), and the values were increased by 8.60%, 22.58%, and 76.11% in leaves, respectively (**Figures 4B, C**).

### 3.5 Effect of MWCNTs on the gene expression of *MhNRTs* in *Malus hupehensis* seedlings

**Figure 5:**
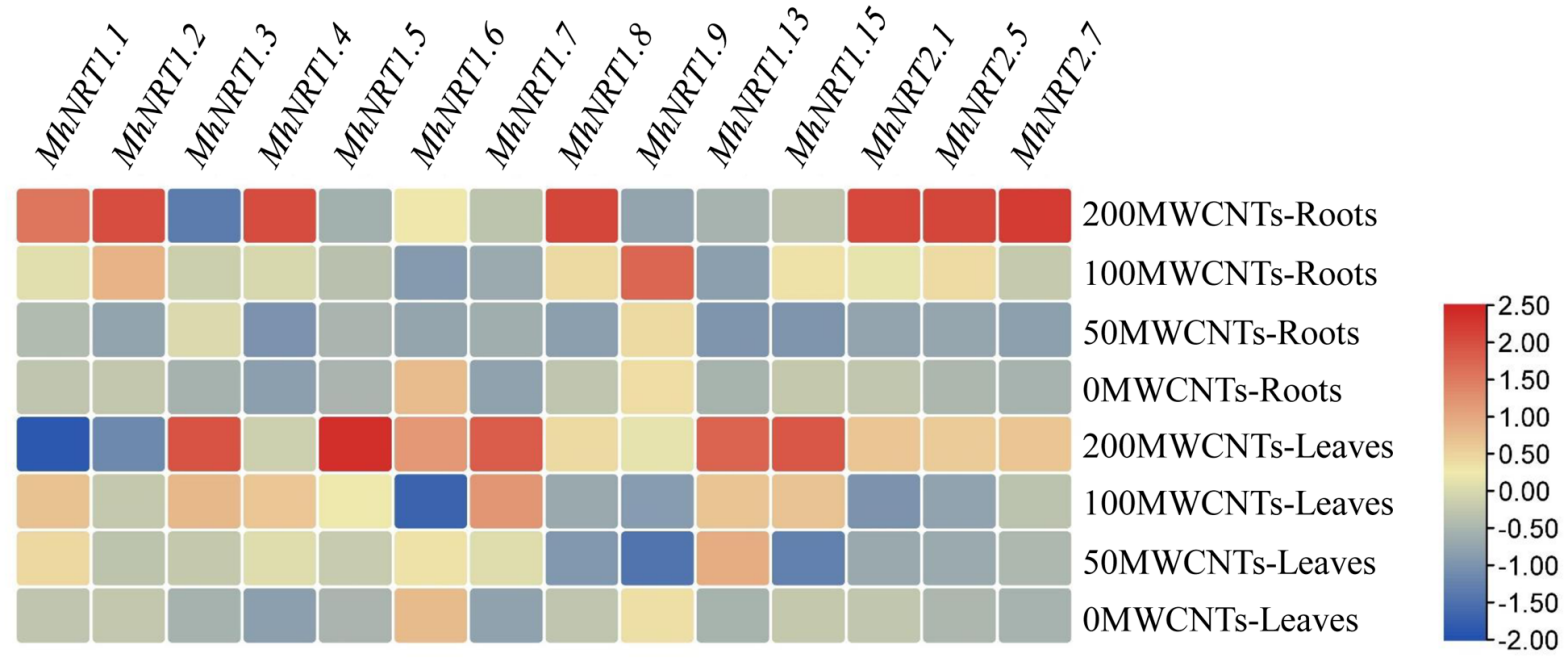
Response of *MhNRTs* expression levels in *Malus hupehensis* seedlings to MWCNTs treatments. For each gene, expression in roots or leaves of plants under the 0 concentration of MWCNTs was defined as 1, and corresponding fold-changes were calculated under other concentrations of MWCNTs. The heatmap was constructed using TBtools.

MWCNTs significantly affected the transcript levels of genes related to nitrate uptake and transport (*P*< 0.05), and the transcript levels could vary among different MWCNTs concentrations, organ types, and *NRTs* types (**Figure 5**). 50 µg·mL^-1^ MWCNTs significantly increased the transcript levels of *MhNRT1.3* and *MhNRT1.7* in *Malus hupehensis* roots but decreased the transcript levels of the other *NRTs* (*P*< 0.05); 100 µg·mL^-1^ MWCNTs significantly increased the transcript levels of 12 *NRTs* genes in roots including *MhNRT1.1*, *MhNRT1.2*, *MhNRT1.3*, *MhNRT1.4*, *MhNRT1.5*, *MhNRT1.7*, *MhNRT1.8*, *MhNRT1.9*, *MhNRT1.15*, *MhNRT2.1*, *MhNRT2.5*, and *MhNRT2.7* (*P*< 0.05); and 200 µg·mL^-1^ MWCNTs significantly increased the transcript levels of 8 *NRTs* genes in roots including *MhNRT1.1*, *MhNRT1.2*, *MhNRT1.4*, *MhNRT1.7*, *MhNRT1.8*, *MhNRT2.1*, *MhNRT2.5*, and *MhNRT2.7* (*P*< 0.05).

MWCNTs at 50, 100, and 200 µg·mL^-1^ significantly increased transcript levels of *MhNRT1.4*, *MhNRT1.5*, *MhNRT1.7*, *MhNRT1.13*, and *MhNRT2.7* (*P*< 0.05), but decreased the transcript levels of *MhNRT1.9* in *Malus hupehensis* leaves. In addition, 50 µg·mL^-1^ MWCNTs also significantly increased the transcript levels of *MhNRT1.1* (*P*< 0.05); 100 µg·mL^-1^ MWCNTs significantly increased the transcript levels of *MhNRT1.1*, *MhNRT1.3*, and *MhNRT1.15* in leaves (*P*< 0.05); and 200 µg·mL^-1^ MWCNTs significantly increased the transcript levels of *MhNRT1.3*, *MhNRT1.6*, *MhNRT1.8*, *MhNRT1.15*, *MhNRT2.1*, and *MhNRT2.5* in leaves (*P*< 0.05).

### 3.6 MWCNTs can be absorbed by *Malus hupehensis* seedlings roots

**Figure 6:**
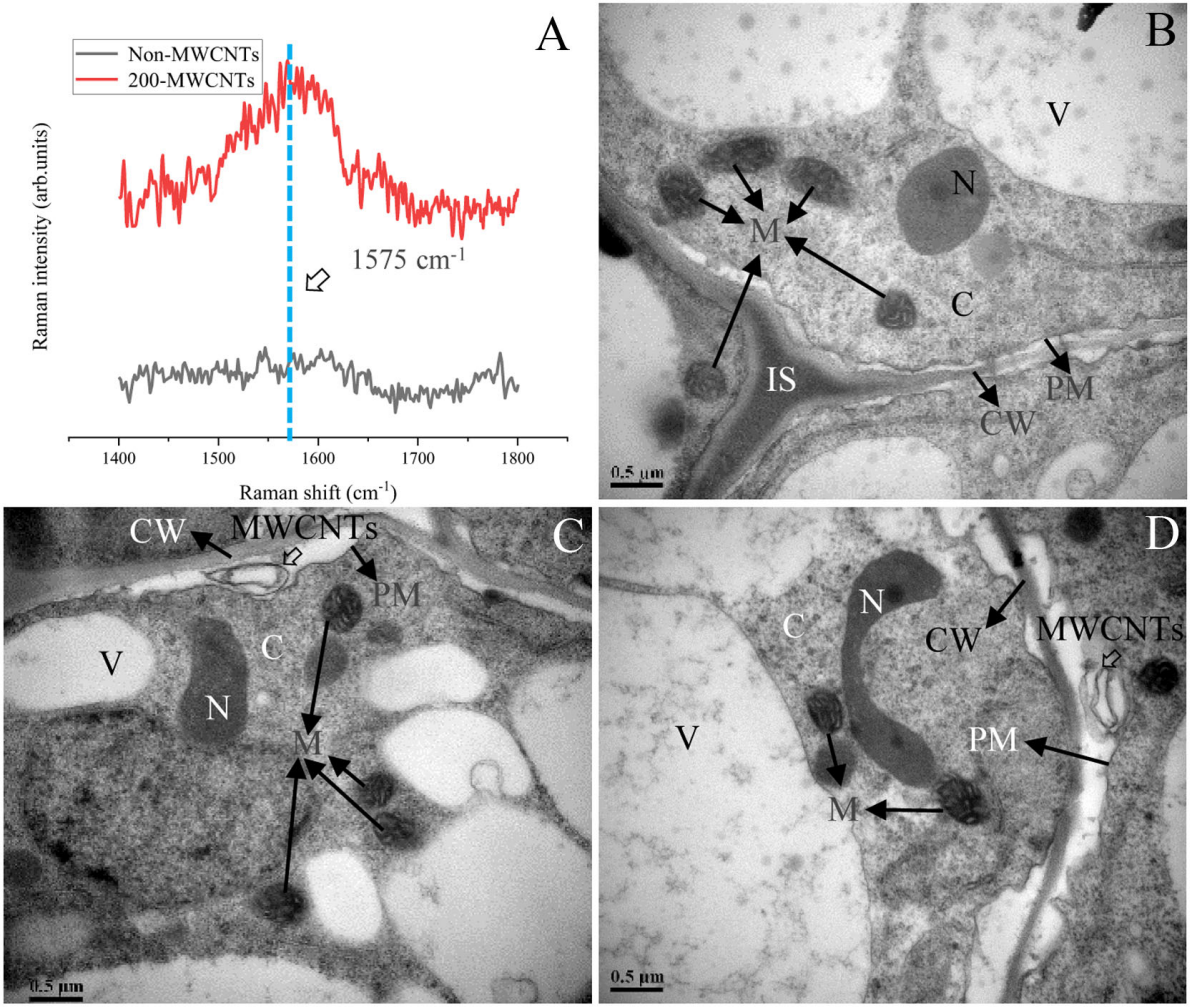
Raman analysis of the *Malus hupehensis* seedlings roots following the control (Non-MWCNTs) and 200 µg·mL^-1^ MWCNTs (200-MWCNTs) treatments **(A)**. TEM images of the *Malus hupehensis* seedlings roots following the control **(B)** and 200 µg·mL^-1^ MWCNTs **(C, D)** for 30 days. Black solid and hollow arrows in **(B–D)** point to organelles and MWCNTs, respectively, scale bars=0.5 µm. CW, cell wall; PM, plasma membrane; IS, intercellular space; N, nucleus; C, cytoplasm; M, mitochondria; V, vacuole.

The Raman analysis indicated the characteristic peak (G band) was between 1492–1650 cm^-1^ (the strongest Raman peak was at 1575 cm^-1^) (**Supplementary Figure 1C**), and the presence of the G band was associated with the existence of the MWCNTs in the plant samples (Khodakovskaya et al., 2012). As shown in **Figure 6A**, the G band was detected in spectra of *Malus hupehensis* seedlings roots samples under the 200 µg·mL^-1^ MWCNTs treatment (200- MWCNTs), but was not detected in control samples (Non-MWCNTs). In addition, Raman analysis did not indicate any similar peak in the spectra of *Malus hupehensis* stems and leaves (**Figure 6A**).

TEM imaging clearly showed the presence of black stripes between the cell wall and cytoplasmic membrane in the roots tissue of *Malus hupehensis* seedlings following the 200 µg·mL^-1^ MWCNTs treatment (**Figures 6C, D**), which was not present in the stems and leaves (**Supplementary Figures 2C, F**). No MWCNTs were observed in the roots, stems, and leaves of control (Non-MWCNTs) plant samples (**Figure 6B**; **Supplementary Figures 2B, E**).

### 3.7 Correlation analysis of root growth indices and stem and leaf fresh weight with nitrate metabolism parameters under MWCNTs treatments

**Figure 7:**
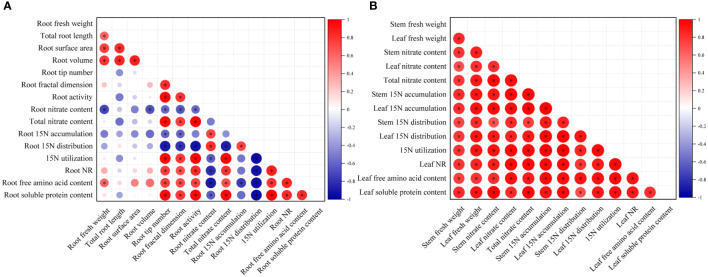
Correlation analysis of root growth indices **(A)**, and stem and leaf fresh weight **(B)** with nitrate metabolism parameters under MWCNTs treatment. Root growth indices indicate root fresh weight, root architecture parameters, and root activity; nitrate metabolism parameters indicate nitrate content, accumulation, and distribution ratio of ^15^N-KNO_3_ in each organ, ^15^N-KNO_3_ utilization ratio, NR activity, free amino acid, and soluble protein content. The red circles in each small square represent positive correlations, and the blue ones represent negative correlations, the depth of the color represents the degree of correlation between two indicators. *Indicate significant difference at *P*< 0.05.

The root growth indices (fresh weight, root architecture parameters, and root activity), stem and leaf fresh weight, and nitrate metabolism parameters (NR activity, the accumulation, distribution ratio, and utilization ratio of ^15^N-KNO_3_, and free amino acid and soluble protein contents), all the above continuous variables were normally distributed with homogenous variances between groups, so a Pearson correlation analysis was carried out to quantify the relationship between the above parameters in roots and aboveground parts, respectively (**Figure 7**).

As shown in **Figure 7**, root nitrate content was significantly and negatively correlated with root volume, root tip number, root fractal dimension, and root activity, and was the most strongly correlated with root fresh weight (*r* = -0.717, *P*< 0.05) (The correlation coefficients among the root indices are shown in **Supplementary Table 3**). The root tip number, root fractal dimension, and root activity were significantly and negatively correlated with the ^15^N-KNO_3_ distribution ratio in roots, while were significantly and positively correlated with the total nitrate content, the ^15^N-KNO_3_ utilization ratio of seedlings, root NR activity, and root soluble protein content, among which the root activity was the most strongly correlated with the ^15^N-KNO_3_ distribution ratio in roots, total nitrate content, ^15^N-KNO_3_ utilization ratio of seedlings, root NR activity, and root soluble protein content, the correlation coefficients were -0.905, 0.978, 0.979, 0.828, and 0.954, respectively (*P*< 0.05). The ^15^N-KNO_3_ accumulation in roots was significantly and negatively correlated with root tip number and root fractal dimension (*r* = -0.594、-0.588, respectively, *P*< 0.05). Root free amino acid content was significantly and positively correlated with root fresh weight, root fractal dimension, and root activity, and was the most strongly correlated with root tip number (*r* = 0.747, *P*< 0.05).

Leaf nitrate content, total nitrate content of seedlings, leaf ^15^N-KNO_3_ accumulation, leaf ^15^N-KNO_3_ distribution ratio, the ^15^N-KNO_3_ utilization ratio of seedlings, NR activity, free amino acid and soluble protein content of seedlings were all significantly and positively correlated with leaf fresh weight, and the correlation coefficients were 0.816, 0.881, 0.921, 0.917, 0.908, 0.850, 0.787, and 0.913, respectively (*P*< 0.05) (The correlation coefficients among the aboveground indices are shown in **Supplementary Table 4**). Stem nitrate content, total nitrate content of seedlings, stem ^15^N-KNO_3_ accumulation, stem ^15^N-KNO_3_ distribution ratio, and the ^15^N-KNO_3_ utilization ratio of seedlings were all significantly and positively correlated with stem fresh weight, and the correlation coefficients were 0.789, 0.777, 0.805, 0.738, and 0.799, respectively (*P*< 0.05) (**Supplementary Table 4**).

## 4 Discussion

Exogenous carbon such as carbonized apple branches can promote the growth and nitrogen utilization of *Malus hupehensis* seedlings (Cao et al., 2019; Shi et al., 2022), however, these materials have a large size range with mostly above microns, and the effects of exogenous carbon on seedling growth and nutrient uptake vary with the particle size. Many of the unique physicochemical properties and biological effects arise from their high-volume-to-surface ratio, and these properties result in an increase in the efficiency of material usage, thus the nanomaterials including MWCNTs have led to a great interest in agriculture and food production (Feregrino-Perez et al., 2018; Ashraf et al., 2021). The nanomaterials show a dramatic dose-dependence of their biological effects, according to the study by Khodakovskaya et al. (2012), 0.1 µg·mL^-1^ MWCNTs inhibited the growth of tobacco cells, while 5–500 µg·mL^-1^ MWCNTs could significantly promote the growth of tobacco cells, and the facilitation was enhanced with the increasing concentration. The increase of aboveground biomass (stems and leaves fresh weight) also showed the same dose-dependent effects following the 50–200 µg·mL^-1^ MWCNTs treatments (**Figure 1G**). MWCNTs could promote the expression of photosynthesis-related genes and improve leaf photosynthetic efficiency in rice (Zhang et al., 2017), also increase the leaf photosynthetic efficiency and chlorophyll content in maize (Hu et al., 2021), the improvement of leaf photosynthetic efficiency is the basic premise for increasing stems and leaves biomass, which may be an important reason for MWCNTs to increase the fresh weight of stems and leaves of *Malus hupehensis* seedlings.

Leaf photosynthetic efficiency requires the maintenance of nutrients and water, plants can improve the water and nutrients uptake by modulating their root growth and architecture (Meier et al., 2020). MWCNTs increased the total root length, root surface area, root volume, root tip number, and root fractal dimension, and also significantly increased the root activity of *Malus hupehensis* seedlings (**Figure 2**). The root tip number can reflect the number of lateral roots of the plant to a certain extent, the formation of lateral roots is an important process for the roots to cope with the ever-changing environment. In addition, lateral roots not only act as physical support but also contributes to water and nutrient uptake for plant growth and development (Cao et al., 2020), meanwhile, lateral roots improve the leaf photosynthetic efficiency and the growth of plants. The root fractal dimension can quantitatively describe the root branching habit and the complexity of the root spatial structure, the greater the value of the fractal dimension, the higher the complexity of the root structure (Walk et al., 2004), the root with complex structure is more developed, and the developed root has a stronger absorption capacity. Root activity is an important reflection of the vital activity and absorption capacity of the roots (White et al., 2018), and the improvement of root activity by MWCNTs would certainly help to promote root nutrient and water uptake. In this way, MWCNTs improved root architecture and root activity, thereby promoting the plant nutrients and water uptake, and improving leaf photosynthetic performance and fresh weight of stems and leaves.

Nitrogen is an essential element for maintaining leaf photosynthesis and promoting plant growth, and nitrate is a nitrogen nutrient that is easily absorbed and used by plants. The results of correlation analysis (**Figure 7**) showed that the nitrate content of seedlings was significantly and positively correlated with root tip number and root fractal dimension (*r* =0.956, 0.755, respectively, *P*< 0.05) (**Supplementary Table 3**), this indicated that the number of lateral roots and root branching ability was important factors in improving nitrate uptake and utilization in *Malus hupehensis* seedlings. In addition, root activity was also highly correlated with the nitrate content of seedlings (*r* =0.978, *P*< 0.05) (**Supplementary Table 3**). The developed root increased the contact area with nutrients, and the increased root activity improved the nutrient uptake capacity of the roots, all of which provided for the efficient uptake and utilization of nitrate. Our results proved that the MWCNTs did increase the nitrate content in *Malus hupehensis* seedlings (**Figure 1H**). Hu et al. (2021) also concluded that MWCNTs could significantly increase the nitrate content of maize seedlings, and we further demonstrated this using the ^15^N isotope tracer techniques, and the accumulation and utilization of ^15^N-KNO_3_ by *Malus hupehensis* seedlings could be increased by up to 85.40% and 85.43%, respectively, within the concentration range of MWCNTs we used (**Figure 3**). Furthermore, nitrate uptake by plants is mainly dependent on nitrate transporters (*NRTs*) (Wang et al., 2012), and our study found that 100 and 200 µg·mL^-1^ MWCNTs induced an increase in the transcript levels of most *NRTs* (*MhNRT1.1*, *MhNRT1.2*, *MhNRT1.4*, *MhNRT1.7*, *MhNRT1.8*, *MhNRT2.1*, *MhNRT2.5*, and *MhNRT2.7*) in *Malus hupehensis* roots (**Figure 5**), this was the intrinsic basis for MWCNTs to promote root uptake and increase the nitrate content in *Malus hupehensis* seedlings.

Interestingly, we found that MWCNTs increased the nitrate content in whole plant of seedlings, but decreased the nitrate content in roots (**Figure 1H**), the results of stable isotope also showed that the distribution ratio of ^15^N-KNO_3_ in roots decreased with the increasing concentration of MWCNTs (**Figure 3B**), this indicated that MWCNTs promoted not only nitrate acquisition by roots but also root to aboveground part nitrate transport. NR is a key enzyme regulating nitrate assimilation and its activity can affect the metabolism of proteins and amino acids (Hu et al., 2016), the increased NR activity in roots and leaves resulted in more accumulation of free amino acids and soluble proteins in the roots and leaves of *Malus hupehensis* seedlings (**Figure 4**). In addition, it has been shown that *AtNRT1.9* is mainly involved in top-down nitrate transport in *Arabidopsis* (Wang and Tsay, 2011), and the results showed that MWCNTs significantly decreased the transcript levels of *MhNRT1.9* in *Malus hupehensis* seedlings leaves (**Figure 5**), thus resulted in a reduction in nitrate transport from the leaves to the roots, which is a reason why MWCNTs reduced the distribution ratio of nitrate in roots.

MWCNTs accumulated between the cell wall and cytoplasmic membrane of Malus hupehensis seedlings roots tissue which was observed by TEM (**Figure 6**), however, MWNCTs were not transported to the aboveground (**Supplementary Figure 2**), this might be closely related to the physicochemical properties, such as size and shape of MWCNTs. It was reported that gold nanospheres (5–20 nm diameter) accumulated at the periphery of the cell plasma membrane/wall of tobacco leaves following infiltration into mature tobacco cells, and the smaller particles associated with plant cell wall faster than their larger counterparts, but no gold nanospheres of any size entered tobacco cells, while the gold nanorods (13 nm × 68 nm) did enter plants cells (Zhang et al., 2022). In addition, nanoparticle carbon (C) 70 could enter tobacco cells and be transported in plants, while MWCNTs could not be taken up by tobacco, which is also due to the difference in material shape (Lin et al., 2009). MWCNTs were likely to stimulate a large number of information receptors on the cytoplasmic membrane, induced the expression of genes such as NRTs in roots through intracellular signal transduction, and then regulated the uptake and transport of nitrate in roots, although MWCNTs only stayed between the cell wall and cytoplasmic membrane of Malus hupehensis roots.

## 5 Conclusion

**Figure 8:**
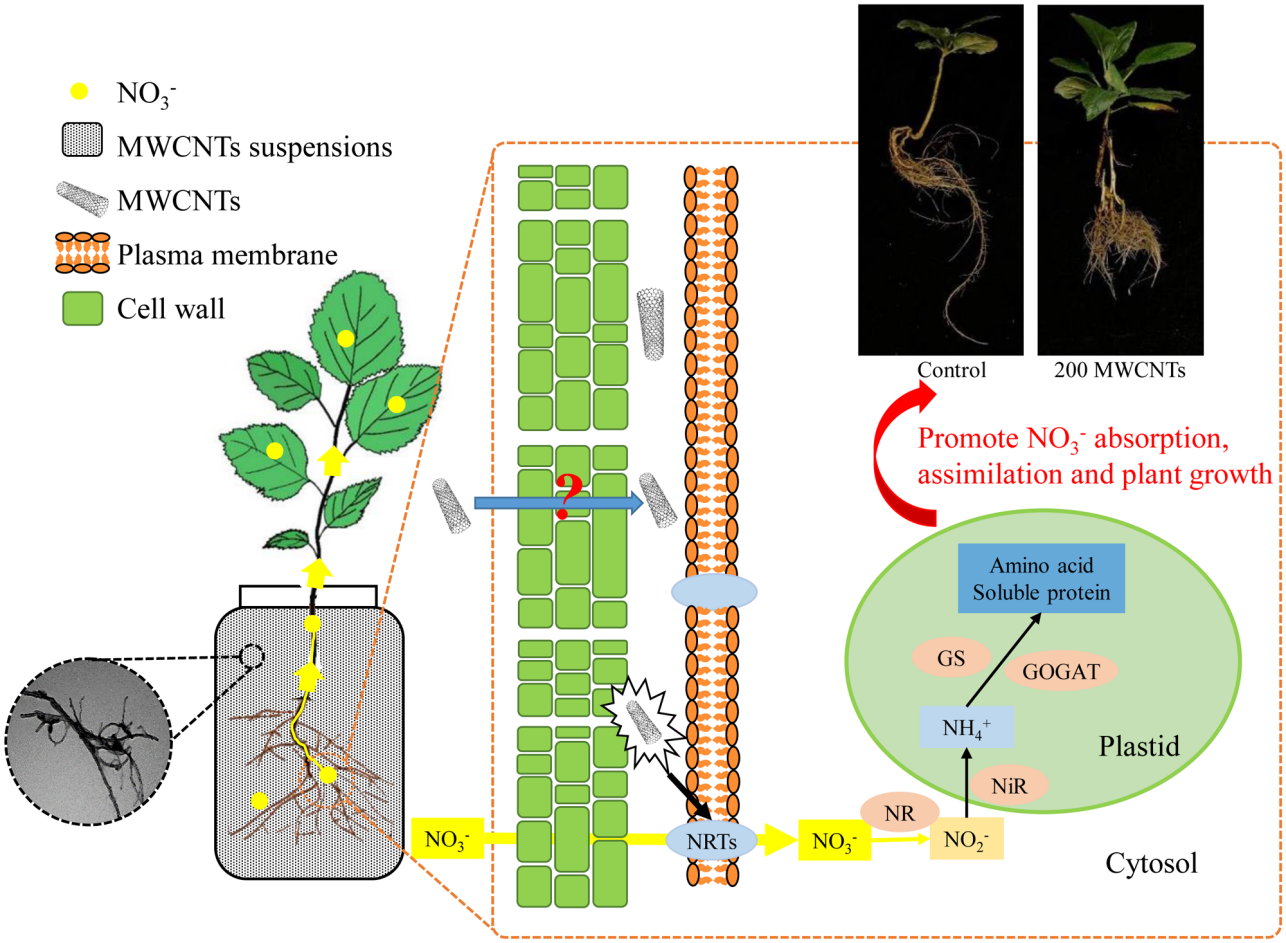
Mechanisms of MWCNTs promoting nitrate uptake and assimilation in roots of *Malus hupehensis* seedlings.

In summary, we employed MWCNTs to investigate their effects on the uptake, distribution, and assimilation of nitrate in *Malus hupehensis* seedlings, and 200 µg·mL^-1^ MWCNTs showed the optimal effects. MWCNTs improved the root growth of *Malus hupehensis* seedlings, promoted the nitrate uptake and transport through entering into the root tissue and stimulating the expression of *MhNRTs*. MWCNTs improved the assimilation of nitrate and increased the accumulation of free amino acids and soluble proteins in seedlings by regulating NR activity, thus promoting the growth of *Malus hupehensis* seedlings (**Figure 8**). Results of correlation analysis showed that root tip number, root fractal dimension, and root activity were the main factors affecting root uptake and assimilation of nitrate.

## Data availability statement

The original contributions presented in the study are included in the article/**Supplementary Material**. Further inquiries can be directed to the corresponding authors.

## Author contributions

HY presented ideas and conducted critical review, commentary or revision. JYS designed the experiments and completed the original draft. MX, JFS, JL and WZ completed proofreading of experiments methods and papers. All authors contributed to the article and approved the submitted version.
