# Supplementary material for: Multi-walled carbon nanotubes promote the accumulation, distribution, and assimilation of 15N-KNO3 in Malus hupehensis by entering the roots
Source: Front Plant Sci. 2023 Mar 9;14:1131978. doi: 10.3389/fpls.2023.1131978 (PMC10033859; doi:10.3389/fpls.2023.1131978)
Supplement: Supplementary file 1 [file DataSheet_1.docx]

Supplementary Material

Multi-walled carbon nanotubes promote the accumulation, distribution, and assimilation of ^15^N-KNO_3_ in *Malus hupehensis* by entering the roots.

**Junyuan Shi^1^, Mi Xun^1^, Jianfei Song^1^, Jiaqi Li^1^, Weiwei Zhang^1*^, Hongqiang Yang^1*^**

^1^College of Horticulture Science and Engineering, Shandong Agricultural University, Daizong Street No. 61, Taishan District, Tai’an, Shandong, 271018, China

*** Correspondence:**
Hongqiang Yang

hqyang@sdau.edu.cn

Weiwei Zhang

zhangww@sdau.edu.cn

# Supplementary Data

Supplementary Material should be uploaded separately on submission. Please include any supplementary data, figures and/or tables.

Supplementary material is not typeset so please ensure that all information is clearly presented, the appropriate caption is included in the file and not in the manuscript, and that the style conforms to the rest of the article.

# Supplementary Figures and Tables

For more information on Supplementary Material and for details on the different file types accepted, please see [here](https://www.frontiersin.org/guidelines/author-guidelines#supplementary-material).

## Supplementary Figures


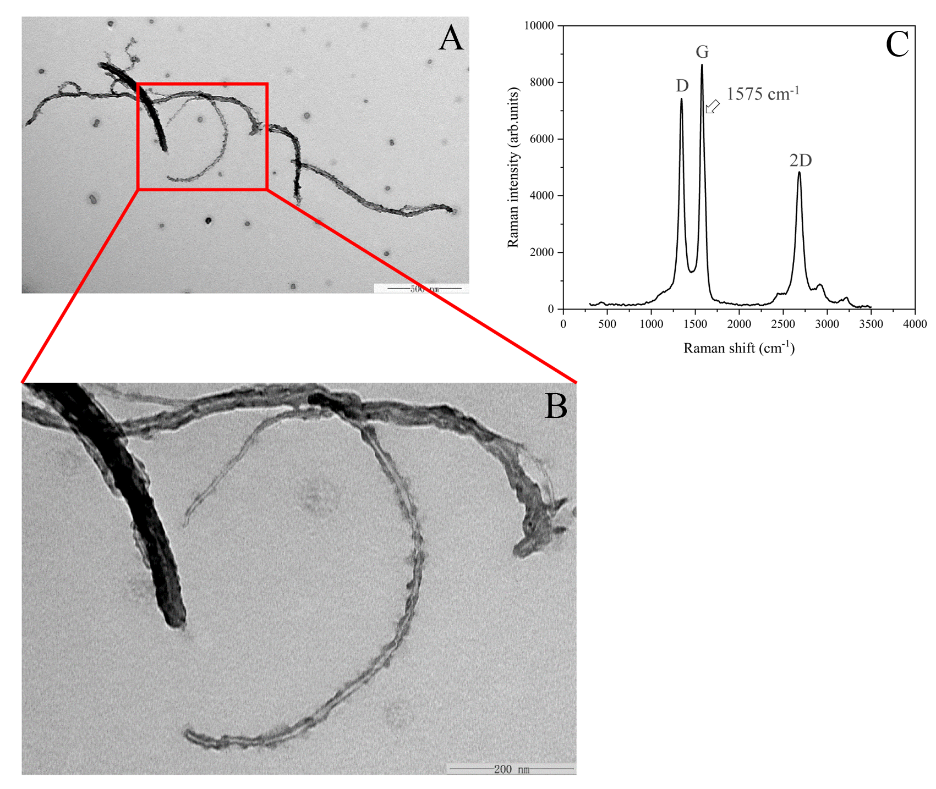


**Supplementary Figure 1. Transmission electron microscopy (TEM) images (A and B) and Raman analysis (C) of MWCNTs used in this study.** (A) Multiple MWCNTs cluster together, scale bars=500 nm; (B) Single MWCNT local magnification image, scale bars=200 nm; (C) The D band is present between 1250–1440 cm^-1^ and is related to the presence of defects and impurities in MWCNTs, the G band is present between 1500-1700 cm^-1^ and is relatively constant for the characteristic peak of MWCNTs; the 2D band is between 2520-2800 cm^-1^ and is associated with the degree of MWCNTs crystallinity.


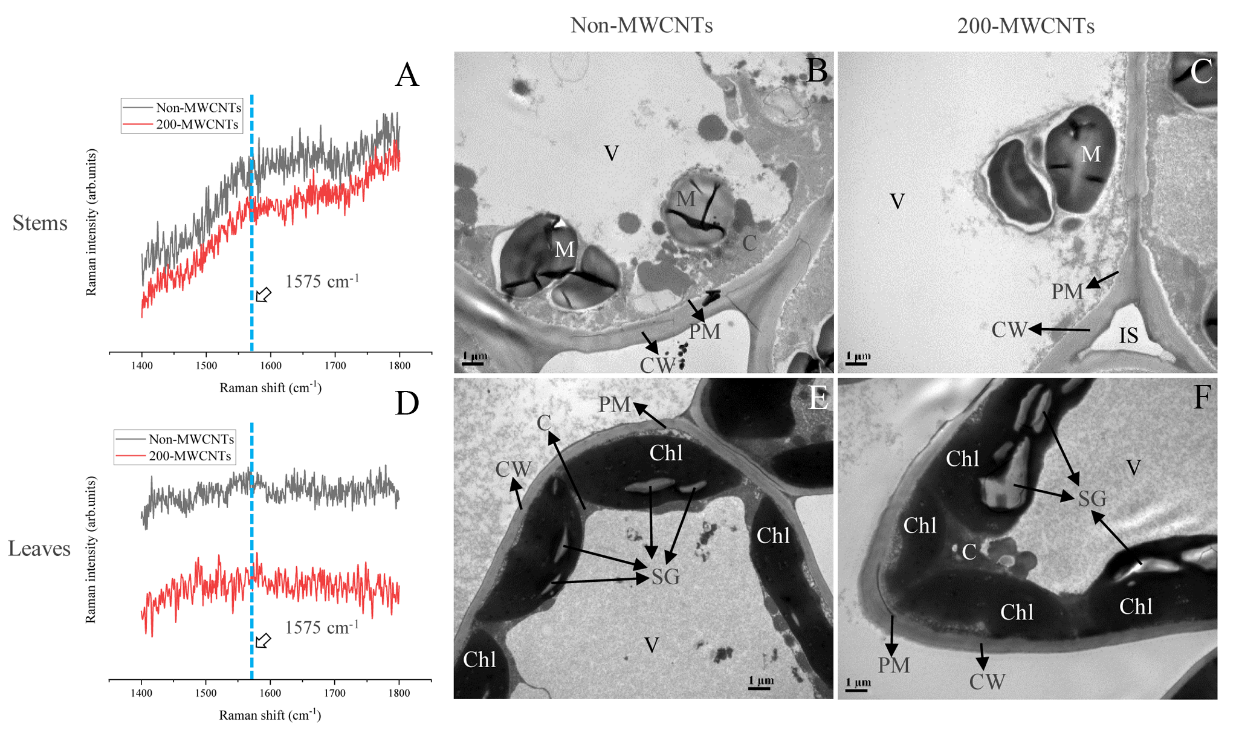


**Supplementary Figure 2. Raman analysis of the *Malus hupehensis* seedlings stems and leaves following the control (Non-MWCNTs) and 200 µg·mL^-1^ MWCNTs (200-MWCNTs) treatments (A and D). TEM images of the *Malus hupehensis* seedlings stems and leaves following the control (B and E) and 200 µg·mL^-1^ MWCNTs (C and F) for 30 days.** Black solid arrows in B, C, E, and F point to organelles, scale bars=1 µm. CW: cell wall; PM: plasma membrane; IS: intercellular space; C: cytoplasm; Chl: chloroplast; SG: starch granule; M: mitochondria; V: vacuole.

## Supplementary Tables

**Supplementary Table 1.** Characterization of MWCNTs

| Project | Parameter |
| --- | --- |
| Purity | >98% (wt%) |
| Inner diameter | 5-10 nm |
| Outer diameter | 10-20 nm |
| Length | 10-30 µm |
| Specific surface area | >150 m^2^·g^-1^ |
| Ash | <1.5% (wt%) |
| Appearance | Black powder |
| Making method | Chemical Vapor Deposition Method |
| Metal content (wt%) | Fe 0.004% |
|  | Co 0.003% |
|  | Ni 0.002% |
|  | Zn 0.001% |
|  | Cu 0.001% |
|  | Mo 0.001% |

Note: The metal content of the MWCNTs was measured using Inductively coupled plasma-mass spectrometry (ICP-MS) (NexION 300X, USA), and all other data in this table are provided by Chengdu Organic Chemistry Co. Ltd.

**Supplementary Table 2.** Primers used for qPCR determination of *MhNRT1* and *MhNRT2* family genes in *Malus hupehensis* seedlings.

| Group | Gene | Forward primer（5’-3’） | Reverse primer（5’-3’） |
| --- | --- | --- | --- |
| *MhNRT1* | *MhNRT1.1* | CCGTTTCTTGGATAAGGCAG | TTGCTTGGGACACTGAGAAT |
|  | *MhNRT1.2* | ATACCGGTGTTTCTTGCTGG | GTGTTTGATTCCGATACCGC |
|  | *MhNRT1.3* | GGTGGTGGTGGGATAAAATC | GCTGATATTCCGTACCCCAA |
|  | *MhNRT1.4* | CTTGCTTTTAACGACCGTGT | AACACGCTAATAGGCAGAGT |
|  | *MhNRT1.5* | CTACCTGGCTATGAACCTCG | CTAAGACTTGGCAAAACCTCC |
|  | *MhNRT1.6* | TACTTGAGCAGTTTGGTGGT | TCTACGTACACATTTGTGGC |
|  | *MhNRT1.7* | TGTGTCTTGCTCCTTTGCC | GATAACGTTGCGCACAAAAC |
|  | *MhNRT1.8* | TGGCTGTGCACAATTCTCTA | TTCCACAGTCTGGCAACAA |
|  | *MhNRT1.9* | CAATCGACGCCTTGGAAAAA | GCATCGTGATCACAGAGAGA |
|  | *MhNRT1.13* | TCAAAATACTGCTTTCAGTTATTCC | AGTGTCGTAGAGAGGGACG |
|  | *MhNRT1.15* | TCCGCTAGGTTCATAATCGG | TAGCGTCCAAGGAAAGAATCA |
| *MhNRT2* | *MhNRT2.1* | GCATTCTTCATTCCAGGGTG | CCCATGGAGTAGCCATAGAG |
|  | *MhNRT2.5* | AGCCCAAAGACAAATTCTCG | AATATTCGCCAACCCGAAAC |
|  | *MhNRT2.7* | GGAACAATGGGGGCGGT | TCAGTTTCTGGTCCGCTTAGA |
|  | *Actin* | TAAGGCTGGATTTGCTGGAG | GCATCTTTCTGACCCATTCC |

**Supplementary Table 3.** Correlation coefficients (*r*) between root growth indices and nitrate metabolism parameters in *Malus hupehensis* seedlings.

|  | RFW | TRL | RSA | RV | RTN | RFD | RA | RNC | TNC | RNA | RND | NU | RNR | RFAAC | RSPC |
| --- | --- | --- | --- | --- | --- | --- | --- | --- | --- | --- | --- | --- | --- | --- | --- |
| RFW | 1.000 |  |  |  |  |  |  |  |  |  |  |  |  |  |  |
| TRL | 0.619* | 1.000 |  |  |  |  |  |  |  |  |  |  |  |  |  |
| RSA | 0.745** | 0.821** | 1.000 |  |  |  |  |  |  |  |  |  |  |  |  |
| RV | 0.796** | 0.856** | 0.873** | 1.000 |  |  |  |  |  |  |  |  |  |  |  |
| RTN | 0.028 | -0.425 | -0.111 | -0.021 | 1.000 |  |  |  |  |  |  |  |  |  |  |
| RFD | 0.226 | -0.160 | 0.029 | 0.266 | 0.811** | 1.000 |  |  |  |  |  |  |  |  |  |
| RA | -0.013 | -0.504 | -0.163 | -0.066 | 0.949** | 0.783** | 1.000 |  |  |  |  |  |  |  |  |
| RNC | -0.717** | -0.219 | -0.499 | -0.661* | -0.594* | -0.658* | -0.580* | 1.000 |  |  |  |  |  |  |  |
| TNC | -0.064 | -0.552 | -0.244 | -0.147 | 0.956** | 0.755** | 0.978** | -0.501 | 1.000 |  |  |  |  |  |  |
| RNA | -0.502 | -0.349 | -0.453 | -0.563 | -0.594* | -0.588* | -0.488 | 0.721** | -0.420 | 1.000 |  |  |  |  |  |
| RND | -0.344 | 0.126 | -0.200 | -0.307 | -0.896** | -0.796** | -0.905** | 0.809** | -0.867** | 0.739** | 1.000 |  |  |  |  |
| NU | 0.086 | -0.420 | -0.082 | 0.012 | 0.953** | 0.772** | 0.979** | -0.636* | 0.979** | -0.525 | -0.943** | 1.000 |  |  |  |
| RNR | 0.334 | -0.145 | 0.161 | 0.310 | 0.746** | 0.684* | 0.828** | -0.809** | 0.781** | -0.527 | -0.915** | 0.875** | 1.000 |  |  |
| RFAAC | 0.611* | 0.161 | 0.493 | 0.575 | 0.747** | 0.707* | 0.724** | -0.898** | 0.685* | -0.756** | -0.931** | 0.799** | 0.851** | 1.000 |  |
| RSPC | 0.139 | -0.341 | 0.051 | 0.074 | 0.953** | 0.761** | 0.954** | -0.634* | 0.952** | -0.567 | -0.944** | 0.977** | 0.828** | 0.840** | 1.000 |

Note: RFW: Root fresh weight, TRL: Total root length, RSA: Root surface area, RV: Root volume, RTN: Root tip number, RFD: Root fractal dimension, RA: Root activity, RNC: Root nitrate content, TNC: Total nitrate content, RNA: Root ^15^N accumulation, RND: Root ^15^N distribution, NU: ^15^N utilization, RNR: Root NR, RFAAC: Root free amino acid content, RSPC: Root soluble protein content. *Indicates significant difference at *P* < 0.05, **indicates significant difference at *P* < 0.01.

**Supplementary Table 4.** Correlation coefficients (*r*) between stem and leaf fresh weight and nitrate metabolism parameters in *Malus hupehensis* seedlings.

|  | SFW | LFW | SNC | LNC | TNC | SNA | LNA | SND | LND | NU | LNR | LFAAC | LSPC |
| --- | --- | --- | --- | --- | --- | --- | --- | --- | --- | --- | --- | --- | --- |
| SFW | 1.000 |  |  |  |  |  |  |  |  |  |  |  |  |
| LFW | 0.840** | 1.000 |  |  |  |  |  |  |  |  |  |  |  |
| SNC | 0.789** | 0.879** | 1.000 |  |  |  |  |  |  |  |  |  |  |
| LNC | 0.693* | 0.816** | 0.802** | 1.000 |  |  |  |  |  |  |  |  |  |
| TNC | 0.777** | 0.881** | 0.956** | 0.937** | 1.000 |  |  |  |  |  |  |  |  |
| SNA | 0.805** | 0.914** | 0.898** | 0.955** | 0.961** | 1.000 |  |  |  |  |  |  |  |
| LNA | 0.801** | 0.921** | 0.940** | 0.930** | 0.975** | 0.992** | 1.000 |  |  |  |  |  |  |
| SND | 0.738** | 0.830** | 0.673* | 0.830** | 0.754** | 0.899** | 0.855** | 1.000 |  |  |  |  |  |
| LND | 0.777** | 0.917** | 0.889** | 0.857** | 0.898** | 0.966** | 0.972** | 0.906** | 1.000 |  |  |  |  |
| NU | 0.799** | 0.908** | 0.923** | 0.956** | 0.979** | 0.996** | 0.997** | 0.858** | 0.957** | 1.000 |  |  |  |
| LNR | 0.790** | 0.850** | 0.862** | 0.944** | 0.945** | 0.957** | 0.954** | 0.789** | 0.896** | 0.967** | 1.000 |  |  |
| LFAAC | 0.756** | 0.787** | 0.807** | 0.925** | 0.890** | 0.958** | 0.935** | 0.905** | 0.921** | 0.947** | 0.911** | 1.000 |  |
| LSPC | 0.832** | 0.913** | 0.990** | 0.829** | 0.964** | 0.916** | 0.951** | 0.695* | 0.895** | 0.938** | 0.896** | 0.818** | 1.000 |

Note: SFW: Stem fresh weight, LFW: Leave fresh weight, SNC: Stem nitrate content, LNC: Leave nitrate content, TNC: Total nitrate content, SNA: Stem ^15^N accumulation, LNA: Leave ^15^N accumulation, SND: Stem ^15^N distribution, LND: Leave ^15^N distribution, NU: ^15^N utilization, LNR: Leave NR, LFAAC: Leave free amino acid content, LSPC: Leave soluble protein content. *Indicates significant difference at *P* < 0.05, **indicates significant difference at *P* < 0.01.
